# Investigation of Drug Cocktail Effects on Cancer Cell-Spheroids Using a Microfluidic Drug-Screening Assay

**DOI:** 10.3390/mi8060167

**Published:** 2017-05-24

**Authors:** Ka I. Au Ieong, Chengpeng Yang, Chin To Wong, Angelie C. Shui, Tom T. Y. Wu, Ting-Hsuan Chen, Raymond H. W. Lam

**Affiliations:** 1 Department of Mechanical and Biomedical Engineering, City University of Hong Kong, Hong Kong, China; evauieong3-c@my.cityu.edu.hk (K.I.A.I.); cpyang2-c@my.cityu.edu.hk (C.Y.); chintwong3-c@my.cityu.edu.hk (C.T.W.); acshui2-c@my.cityu.edu.hk (A.C.S.); wu_tsz_yuen@hotmail.com (T.T.Y.W.); thchen@cityu.edu.hk (T.-H.C.); 2 City University of Hong Kong Shenzhen Research Institute, Shenzhen 518057, China; 3 Centre for Biosystems, Neuroscience, and Nanotechnology, City University of Hong Kong, Hong Kong, China

**Keywords:** microfluidic, toxicity, cell, cancer, drug screening

## Abstract

Development of drugs based on potential anti-cancer chemotherapeutic agents has been hindered by its necessary tedious procedures and failure in the clinical trials because of unbearable toxicity and extremely low clinical efficacy. One of the technical challenges is the mismatch between laboratory settings and human body environments for the cancer cells responding upon treatments of the anti-cancer agents. This major limitation urges for applying more reliable platforms for evaluating drugs with a higher throughput and cell aggregates in a more natural configuration. Here, we adopt a microfluidic device integrated with a differential micromixer and multiple microwell-containing channels (50 microwells per channel) for parallel screening of suspending cell spheroids treated by drugs with different combinations. We optimize the culture conditions of the surfactant-coated microwells in order to facilitate the spheroid formation of the breast cancer cell line (MDA-MB-231). We propose a new drug cocktail combined with three known chemotherapeutic agents (paclitaxel, epirubicin, and aspirin) for the drug screening of the cancer cell-spheroids. Our results exhibit the differential responses between planar cell layers in traditional culture wells and cell-spheroids grown in our microfluidic device, in terms of the apoptotic rates under treatments of the drug cocktails with different concentrations. These results reveal a distinct drug resistance between planar cell layers and cell-spheroids. Together, this work offers important guidelines on applying the cell-spheroid microfluidic cultures for development of more efficacious anticancer drugs.

## 1. Introduction

In anticancer drug development, ~90% of the drug candidates fail in clinical trials per year due to intolerable toxicity and low efficiency [1,2]. One of the main culprits for these failures is the use of the planar cell layers in conventional wells for in vitro drug screening. Due to the difference in the cellular microenvironment, the corresponding results often differ drastically from the in vivo responses [3], which urges for an efficient and reliable drug test platform to more closely emulate the in vivo cancer cell behaviors for improving the development of anticancer drug treatments.

‘Three-dimensional’ cell culture techniques [4], including growing tumor spheroids [5,6,7], have been developed in the hopes of solving the problems in drug screening and development [8,9,10,11,12]. It has been shown that cell-spheroids offer physiologic parameters mimicking the in vivo scenarios [3]. For example, the necrotic core and a radial chemical concentration gradient in the spheroids [13] provide complex multicellular structures and mass transport barriers more realistic than monolayer cultures. Thus, such cell aggregating structures could give a better prediction on drug–tumor interactions such as gene expressions [14], ligand responses [15], morphological and molecular aspects [16], and potentially reflect the tumor responses upon chemotherapeutic agents more accurately. Conventional 3D culture techniques for forming spheroids include the hanging-drop method, the non-adherent culture wells, and the bioreactors–spinner flasks [17]. However, these methods require significant biopsy quantities, limiting the number of tests to be performed in the drug screening process.

Recently, increasing attention is being focused on the microfluidic 3D culture platforms for drug-screening assays that require very low concentrations of chemotherapeutic agents and cells [18,19,20]. Earlier studies by Toh et al. [21] and Ong et al. [22] developed a gel-free 3D culture system to reaffirm the significant differences between planar and 3D cultures in microfluidics. Later, Ruppen et al. [23] developed a microfluidic platform using a spheroid model to predict more representative in vivo behaviors of tumor cells, suggesting that microfluidic systems for cell-spheroid culture can be considered as an in vitro tumor model and an anti-cancer drug screening platform [22]. Based on this principle, more technical aspects were further refined. Wu et al. [24] utilized a U-shaped chamber array to trap MCF-7 cells and generate small spheroids that can distinguish between the early or metastatic stage of tumor cells. In addition, Liu et al. [25] demonstrated that microfluidics can offer high-throughput 3D cell culture over a long-term culture period. Hsiao et al. [26] developed a microfluidic platform capable of forming spheroids composed of multiple cell types (cancer cells, osteoblasts endothelial cells) as a drug-screening model. McMillan et al. [27] reported an integrated droplet microfluidic platform that supported spheroid culture in either media or gel scaffold for high throughput anticancer drug screening application. Importantly, Patra et al. [28] reported an important microfluidic technique to form multiple spheroids with a uniform size, which greatly improved consistence of the spheroid properties for more promising results. Later, Patra et al. [29] further demonstrated the drug-screening with flow cytometry analysis, which is a big step toward the pharmaceutical and clinical implementation. Altogether, microfluidics are capable of implementing an efficient and reliable platform for toxicity tests and drug screening.

While the microfluidics enable drug screening on the cancer cells, the personalized therapy may be also achieved by optimizing the concentrations of individual chemotherapeutic agents in cocktails. This drug cocktail is to reduce the necessary levels of the individual agents for higher efficacy and weaker side effects. For breast cancers, the known approved chemotherapeutic agents include epirubicin hydrochloride (also known as epirubicin) and paclitaxel [30]. Paclitaxel-epirubicin (PE) has been extensively studied and become a clinically approved drug cocktail as a first line chemotherapy for metastatic breast cancer [31]. Among other possible breast cancer drug candidates, aspirin, a commonly used agent for pain, fever and inflammation, was recently found to be able to decrease the viability of breast cancer cells [32]. Notably, the lower risk of cancer recurrence at other body sites [33] suggests the possibility of forming the paclitaxel-epirubicin-aspirin as a new drug cocktail, yet the corresponding dosage has to be investigated in detail [34].

In this research, we report a microfluidic assay for parallel drug screening on cancer cell-spheroids. We chose the single agents paclitaxel, epirubicin, and aspirin to form a three-drug cocktail. In particular, we examined the cell growth via the spheroid size increments and cell viability tests, comparing to the results performed with the planar cell cultures. Together, these results revealed the difference between cell layers and spheroids on their desired apoptotic responses under the three-drug cocktail, and further demonstrated the applicability of the device architecture as a parallel drug screening platform using cell-spheroids for more promising anticancer drug cocktail development and optimization.

## 2. Materials and Methods

### 2.1. Device Fabrication

**Figure 1:**
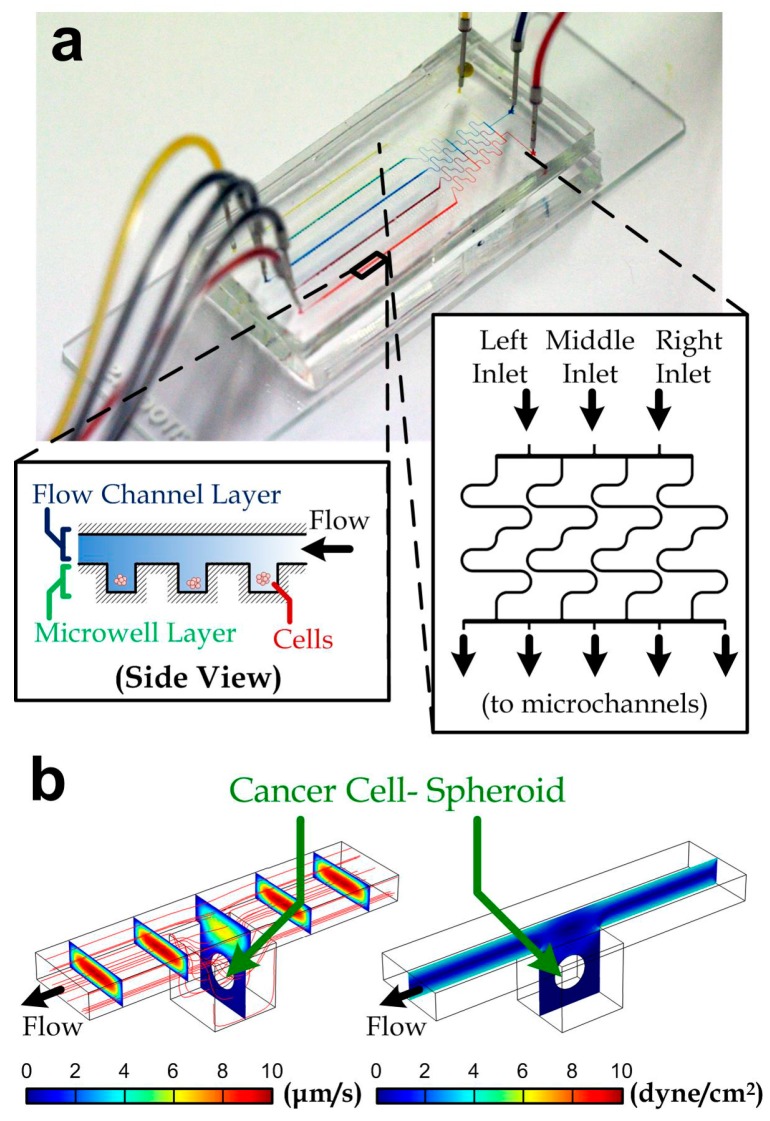
(**a**) A fabricated microfluidic chip for drug-screening assay. Three inlets are shown on the right-hand side with red, blue, and yellow dyes infused via a syringe pump. Five different colors appeared at culturing channels. Insets: side view of the microwell regions along a micro channel (*left*) and geometry of a differential mixer (*right*); (**b**) velocity (*left*) and shear stress (*right*) profiles along a microwell unit containing a cell-spheroid with a diameter of 100 µm. The *red* lines are streamlines.

The device consisting of two layers of microstructures was fabricated by the soft lithography of polydimethylsiloxane (PDMS; Sylgard 184 silicone elastomer, Dow Corning, Midland, MI, USA) [35] mixed with a 10% weight ratio of the curing agent. The upper microstructures contain mainly flow channels and the lower microstructures are the microwells located along the flow channels (*left* inset of Figure 1a). The two molds for the microstructures are both micropatterned photoresist (SU-8 2100, MicroChem, Westborough, MA, USA) on silicon wafers, treated with (tridecafluoro-1,1,2,2-tetrahydrooctyl)-1 trichlorosilane after the fabrication. After molding PDMS for both the structural layers, the upper layer is punched with holes for the drug/medium inlets and outlets. The PDMS layers are then bonded together using oxygen plasma treatment (PDC-32G-2, Harrick Plasma, New York, NY, USA). The combined PDMS substrate is then bonded onto a glass slide using oxygen plasma treatment again for the physical support. The device was then flushed with a surfactant (Pluronics F-127, Thermo Fisher Scientific, Waltham, MA, USA). A fully assembled device is shown in Figure 1a.

### 2.2. Cell Culture

Human MDA-MB-231 breast cancer cells (cat# 92020424, Sigma-Aldrich, St. Louis, MO, USA) were cultured in DMEM/F12 (cat# D6421, Sigma-Aldrich) supplemented with 10% fetal bovine serum and 1% penicillin. The cells were cultured in an incubator with a humidified and 5% CO_2_ environment at 37 °C, and were passaged once they reached 80–90% confluence in the culture wells.

### 2.3. Cell Seeding and Culture on a Chip

We prepared a MDA-MB-231 cell sample in fresh media at a density of 1 × 10^6^ cell/mL. After injecting the cells into the device, we cultured the cells by placing the device with tubing in an incubator (37 °C and 5% CO_2_) for 1 h such that some cells can sink into microwells along the device microchannels. We then flow pure fresh media along the device to flush away cells outside the microwells. We then apply continuous media flow driven by a syringe pump at a flow rate of 300 µL/min overnight for cell aggregation and cell-spheroid formation. Afterward, culture media containing defined drug concentrations were then applied to the device throughout the culture experiments. The device was maintained in the incubator except that it was temporarily transferred to a microscope for imaging at selected time points. For the cell apoptosis tests, we applied a fluorogenic substrate (NucView 488 Caspase 3 Substrate, Biotium, Fremont, CA, USA) to indicate the activity of caspase-3 for the downstream apoptosis events of the cancer cells through the drug treatments.

### 2.4. Flow Simulation

We utilized commercial software (Multiphysics 5.0, COMSOL, Burlington, MA, USA) to analyze the flow profile and the level of shear stress around cell clusters. We constructed a model of a microchannel (length: 500 μm; width: 100 μm; height: 50 μm) and one microwell (width: 100 μm; depth: 100 μm) containing a cell spheroid (diameter: 50 μm) located at the channel center. All the model surfaces set with the no-slip boundary conditions, except that the channel inlet was set with a uniform entering flow rate of 60 µL/min and the channel outlet was set with an open channel with a zero gauge pressure. Because of the viscous flow with a low Reynolds number (*Re* < 1) of our microfluidic platform, the flow should be fully developed within the inlet channel before entering the microwell region. After running the simulation, we examined the shear stress profile around the cell spheroid.

### 2.5. Statistics

Error bars in plots are standard errors. An error bar is not shown for a data point in plots if the error bar is smaller than the data point symbol. We compared two groups of data for any significant difference using a Student’s two-tailed, unpaired *t*-test to obtain the *p*-values. An asterisk in a figure represents a significant difference between two data groups (*p* < 0.05).

## 3. Results and Discussion

### 3.1. Device Design

We adopted a well-established, microfluidic screening strategy for quantifying viability of cancer cell-spheroids under treatment of drug cocktails. We fabricated a microfluidic device consisting of a differential concentration generator [36,37] (inset of Figure 1a) and five downstream microchannels (width: 100 μm; height: 50 µm). The differential concentration generator consisted of four long microchannels between the device inlets and the downstream channels. The long channels were diffusion dominant and achieved thorough mixing at the channel outlets flowing with both the inlet liquids. In principle, all of the inlet liquids were driven by the same syringe pump and therefore the inlet flow rates were the same. The liquid concentrations along the five downstream channels corresponding to the three inlet liquids (left, middle and right) were then (100% (*left* liquid), 0% (*middle* liquid), 0% (*right* liquid)), (50%, 50%, 0%), (17%, 66%, 17%), (0%, 50%, 50%) and (0%, 0%, 100%) as indicated in inset of Figure 1a (*left* to *right*). As a demonstration of the mixing, we have injected color dyes (*red*, *blue* and *yellow*) into the device inlets and the mixed colors (*purple* and *green*) could be observed in the intermediate microchannels (mixer outlets 2 and 4, Figure 1a). Hence, increased drugs combinations can be obtained at the downstream microchannels. The microchannels were constructed with two layers of microstructures: an upper flow channel layer and the lower microwell layer, which has an extra depth of 100 μm in every microwell region (100 μm × 100 μm) as shown in Figure 1b. In the device used in this work, each microchannel contains 50 microwells, and all of the inner surfaces were coated with a surfactant (Pluronics F-127; cat# 9003-11-6, Sigma, St. Louis, MO, USA) for avoiding any cell attachments. Hence, we expect that the seeded cancer cells in the microwells will form cell spheroids due to the cell aggregation and continuous proliferation.

To study the influence of shear stress around cell spheroids, we performed simulation in the microwells. Here, we considered the diameter of the cell spheroid to be 50 µm; and we set the 60 µL/min flow rate along one microchannel as adopted in the practical experiments. The simulated velocity profile of the media/drug flow along a microwell region is shown in Figure 1b (*left*). The shear stress profile (Figure 1b, *right*) indicates that the maximum shear stress over the cell surface (0.083 dyne/cm^2^) is negligible for any known stimulated cell responses [38,39]. Procedures of the device operation are described in Section 2.

### 3.2. Selection of Drug Cocktails Based on Planar Cultures

**Figure 2:**
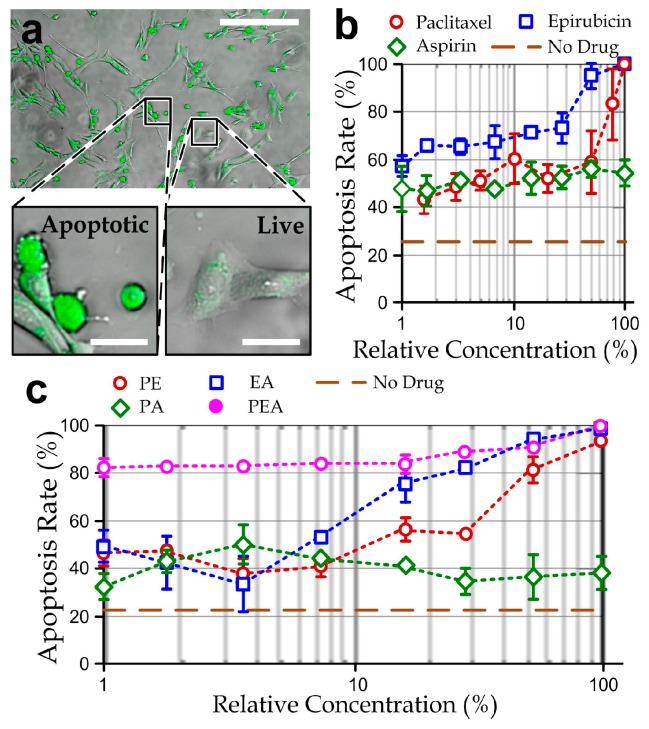
(**a**) fluorescent micrograph showing apoptotic MDA-MB-231 cells (green) treated with 30 nM of paclitaxel for 30 h. Scale bar: 200 µm. Insets: Enlarged micrographs of apoptotic and live cells (scale bar: 25 μm); (**b**) apoptosis rates of MDA-MB-231 cells in traditional culture plates treated by different relative concentrations of single drugs: paclitaxel (100% = 1 μM), epirubicin (100% = 6.4 μM) or aspirin (100% = 32 μM); (**c**) apoptosis rates of the cells treated by different relative concentrations of the two-drug mixtures: PA (100% = paclitaxel: 500 nM; aspirin: 16 µM), EA (100% = epirubicin: 3.2 μM; aspirin: 16 µM), and PE (100% = paclitaxel: 500 nM; epirubicin: 3.2 μM); and the ‘paclitaxel-epirubicin-aspirin (PEA)’ three-drug cocktails (100% = paclitaxel: 333 nM; epirubicin: 2.1 μM; aspirin: 11 µM). *N* > 200 for each of the three repeated experiments for every data point.

The efficacy of the therapeutic agents, paclitaxel, epirubicin, and aspirin [40] was first studied based on cultured cancer cells in conventional well-plates. We first counted the apoptotic MDA-MB-231 cells in the population after the treatment of one selected therapeutic agent with different concentrations (paclitaxel (P): 15 nM–1 µM, epirubicin (E): 50 nM–6.4 µM, or aspirin (A): 100 nM–32 µM) for 48 h. The cells were stained with green fluorescence signals on their cell bodies based on the caspase-3 activities (NucView 488 Caspase 3 Substrate, Biotium, Fremont, CA, USA) as described in Figure 2a. We estimated the apoptosis rate of cells in a culture well by quantifying the stained area relative to the entire cell population. Our results (Figure 2b and Figure S1) indicate that the cell viability reduced with the higher drug concentrations and longer treatment durations of paclitaxel or epirubicin, whereas aspirin cannot cause a majority of cell death in the culture even with a high concentration of 32 µM for 30 h.

Next, we performed the apoptosis tests again for the treatment of two-drug mixtures 0.5 μM paclitaxel/3.2 μM epirubicin (PE), 5 μM paclitaxel/16 μM aspirin (PA) and 3.2 μM epirubicin/16 μM aspirin (EA) with different relative concentrations, defined as the percentage of drugs relative to these drug mixtures, for 48 h. In these experiments, the concentration range of either therapeutic agent in the two-drug mixtures was halved, compared to the single-drug treatments. Our results (Figure 2c and Figure S2) showed that EA and PE drug mixtures can cause similar effects of cancer cell death; and PA can induce only partial cell death to the cell populations. Hence, epirubicin should take an important role in causing apoptotic effects of the cancer cells. It should be noted that the EA and PE drug mixture have an equivalent toxicity of the single-drug treatment of either paclitaxel or epirubicin, implicating the possibility of designing a paclitaxel-epirubicin-aspirin (PEA) drug cocktail with a much lower concentration of the individual agents. To explore this possibility, we repeated the experiments with different relative concentrations of a PEA drug cocktail (0.33 μM paclitaxel/2.1 μM epirubicin/10 μM aspirin, which is only one-third of the max concentration of single agents). Despite the fact that the apoptosis staining dye may induce additional cytotoxicity, these results (Figure 2c and Figure S3) indicate that the PEA cocktail is a potential candidate for breast cancer chemotherapy with high efficacy. In particular, >80% apoptotic cells in the population can be achieved with only the very low concentration of PEA (P: 3.3 nM; E: 21 nM; A: 100 nM) for 30 h of the drug treatment.

### 3.3. Formation of Cancer Cell-Spheroids

**Figure 3:**
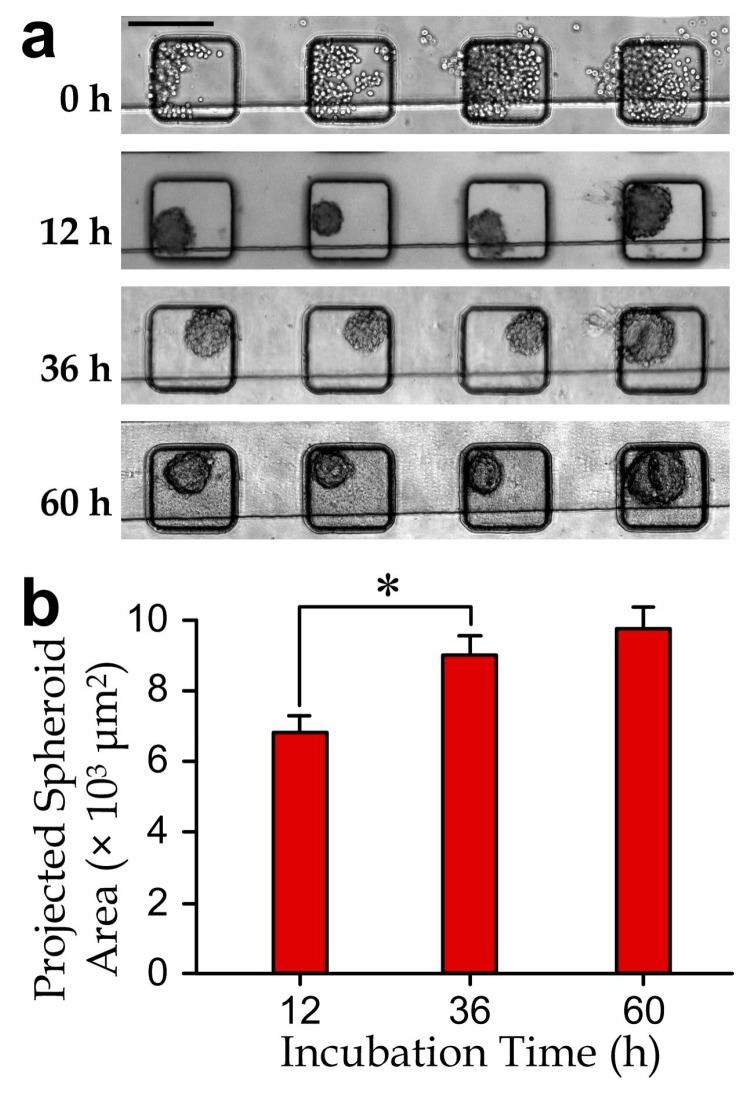
(**a**) Micrographs of the cell-spheroids in a microchannel section during a 60 h culture period. Scale bar: 200 µm; (**b**) projected area increment of cell-spheroids (*N* > 100 for each bar). The asterisk indicates a significant difference with the *p*-value < 0.05.

We performed experiments to validate formation of the cancer cell-spheroids in the microwells by seeding MDA-MB-231 cells into the microfluidic device (Figure 3a), following the well-established procedures as previous research works [5,41,42]. Individual MDA-MB-231 cells (as observed in the microwells after the cell seeding step (day 0)) aggregated as cell-spheroids (day 1) during the first day of cultivation. The cell-spheroids kept growing over the following culture period (day 2 and day 3). Results (Figure 3b) indicate a continuous increment of the project spheroid areas as well as overall growth of the cell populations for at least three days. Hence, we adopted the cell-spheroids formed for two days in the device to test effects of the selected drug cocktails.

### 3.4. Effects of Drug Cocktails on Cancer Cell-Spheroids

**Figure 4:**
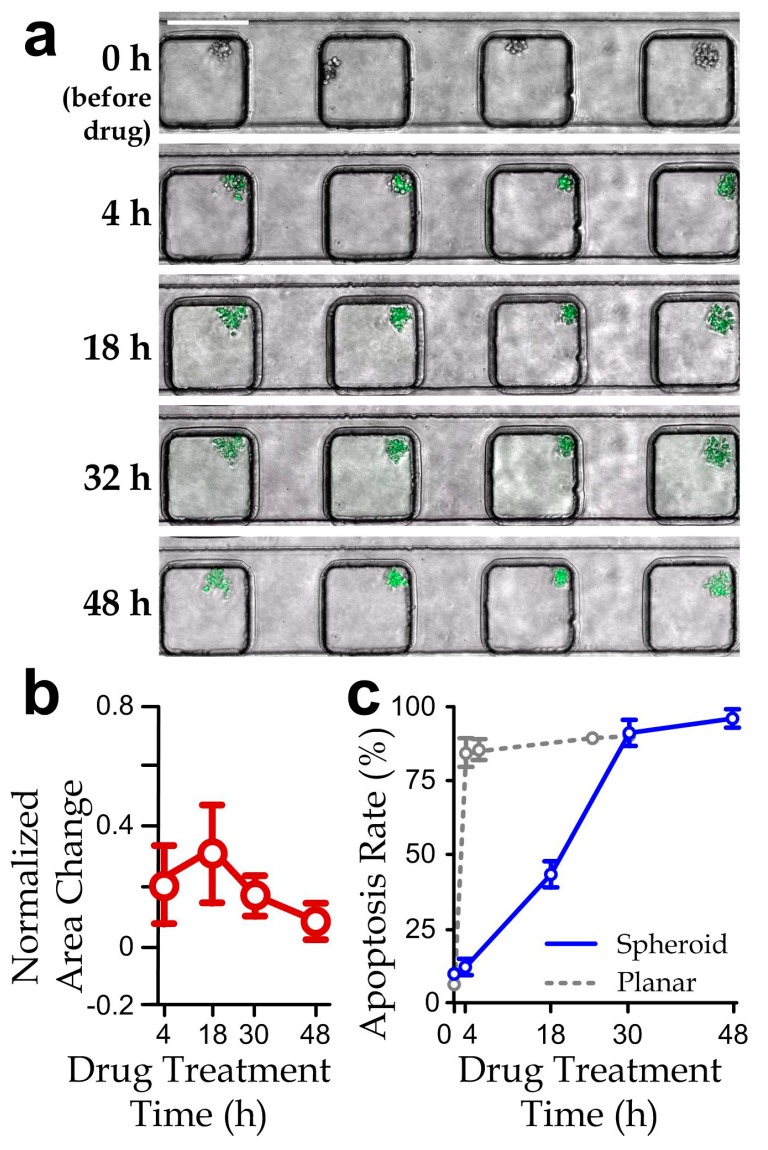
(**a**) Fluorescence micrographs (scale bar: 200 µm); (**b**) normalized projected area variation (relative to the area at 0 h); and (**c**) apoptosis rate of cancer cell-spheroids along a channel section treated by the PEA drug cocktail (paclitaxel: 167 nM; epirubicin: 1 µM; aspirin: 5 µM) over 48 h. *N* > 30 (The results of planar culture extracted from Figure S3 are included here for comparison).

We next implemented the drug tests using the microfluidic device to study the responses of cancer cell-spheroids. The spheroids (projected area: (6.9 ± 0.43) × 10^3^ µm^2^) were first formed inside the device by culturing overnight, where individual cells were pretreated with the apoptosis-labeling fluorescence dye. We tested the PEA cocktail (167 nM paclitaxel, 1 μM epirubicin and 5 μM aspirin, which is 50% of the maximum concentration of PEA cocktail tested in Figure 2c) for 48 h. Notably, the culture duration of spheroids can affect cell viability, including insufficient nutrient supply in the inner spheroid bodies. For instance, Ivascu et al. [43] reported that viability of MDA-MB-231 spheroids can maintain with >95% within 3 days but drop significantly after 10 days. As we chose 48 h as the treatment period as also adopted by Patra et al. [29], any measured reduction in viability comparing to the control case should be mainly caused by the drug treatments. The result showed an insignificant change of the projected spheroid area, suggesting that cell proliferation could be effectively suppressed by the drug treatment (Figure 4b). In addition, we quantified the apoptosis rate of a cell-spheroid as the number of apoptotic cells related to the total cell number in the spheroid. A >85% apoptosis rate of the cell-spheroids was achieved after the treatment (Figure 4c). Remarkably, the drug response of the spheroids appeared to be slower than that of the planar cell layers, e.g., an ~80% apoptosis rate of planar cell layers can be obtained for only 4 h of the drug treatment (Figure S3 and Figure 4c, *hidden line*).

**Figure 5:**
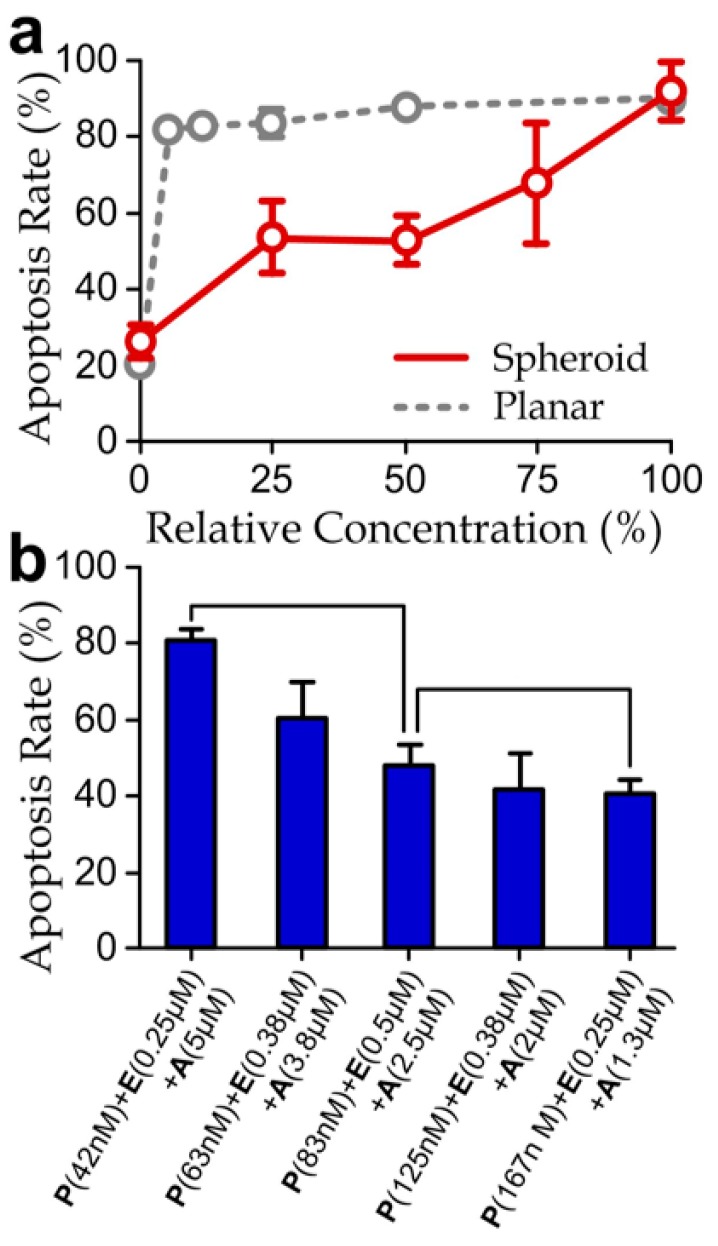
(**a**) Apoptosis rates of cancer cell-spheroids treated different relative concentrations of the drug cocktails (100% = P: 167 nM; E: 1 µM; A: 5 µM) for 48 h. (The results of planar culture extracted from Figure 2c are included here for comparison.) Asterisks indicate significant differences comparing to the planar cell layers; (**b**) apoptosis rates of the spheroids treated with different drug agent combinations for 48 h. *N* > 30.

In addition, we have quantified apoptosis rates of the spheroids treated with different concentrations of the selected PEA cocktail. We applied the micromixer in the device to generate the different drug concentrations, by supplying the left and right device inlets with one side pure media and the other side the 100% PEA cocktail while blocking the middle device inlet. Thus, we could obtain the five-microwell channels with relative concentrations of the PEA cocktail 0%, 25%, 50%, 75% and 100%. The measured apoptosis rates of the drug-treated spheroids were significantly lower than that of the planar cell layers (Figure 5a), reflecting a higher drug resistance of the spheroids. In fact, it has been reported that the higher resistance of the spheroids to chemotherapeutic treatments could be induced by the larger cell–cell contacts, higher cadherin expressions and increased paracrine signaling [44,45,46]. Therefore, optimal doses of the selected PEA cocktails are different between cell layers and spheroids.

We next examined apoptosis rate upon the variation of the concentrations of individual agents in the PEA cocktail. In the screening experiments, we injected three PEA cocktails with different agent concentrations (42 nM (P) + 0.25 μM (E) + 5 μM (A), 83 nM (P) + 0.5 μM (E) + 2.5 μM (A) and 167 nM (P) + 0.25 μM (E) + 1.3 μM (A)) to the inlets of the microfluidic device. Our results (Figure 5b) indicate that an increased partial concentration of aspirin and reduced concentrations of paclitaxel would induce a higher apoptotic rate of the cell-spheroids. In contrast, the change of the concentration of epirubicin did not show significant and consistent influence. Furthermore, the drug concentrations used in this experiment (42 nM (P) + 0.25 μM (E) + 5 μM (A)) can induce a comparable apoptotic rate as the drug with the same aspirin concentration but lower paclitaxel and epirubicin concentrations as previously shown in Figure 5a (the case with 100% relevant drug concentration). Surprisingly, this trend reveals the dominating apoptotic effects caused by aspirin, rather than epirubicin, as shown previously by the planar culture experiments (Figure 3). Considering the molecular weights of epirubicin (544 g/mol), paclitaxel (854 g/mol) and aspirin (180 g/mol), a possible explanation is that the lowest molecular weight and the corresponding highest diffusivity [47] of aspirin imply the deeper penetration of this agent into the cell-spheroid bodies. Considering that it is widely believed that cancer cell-spheroids should provide more representative responses of tumors upon drug treatments than the cell layer [22,25,26,48], this finding suggests that diffusion of drugs throughout the spheroid is likely another key factor to determining drug resistance of the cancer cell-spheroids.

Collectively, our microfluidic device has demonstrated the quantification of apoptotic rates of cell-spheroids under treatments of different drug cocktails. The micromixer design can be further extended to provide more concentration levels with the increased number of mixer outlets and flow microchannels. Further development and applications of this microfludic device will enable more detailed characterization of cancer-cell spheroids and extensive drug screening toward the personalized design of efficacious drugs for cancer patients.

## 4. Conclusions

In this study, a microfluidic drug-screening assay has been successfully fabricated and applied to study cell-spheroids on their resistances to the paclitaxel-epirubicin-aspirin (PEA) drug cocktails. We have conducted simulation on the flow along a microwell section containing a cell-spheroid to verify the negligible shear stress generated around the spheroids. We also performed experiments to confirm the growth of cell-spheroids in the device. Furthermore, we have designed a three-drug cocktail (paclitaxel: 167 nM; epirubicin: 1 µM; aspirin: 5 µM) based on the traditional, planar cell culture in well plates and applied this cocktail with different relative concentrations for screening the apoptosis rates of the cell-spheroids formed in the microfluidic device. Our results have shown that the planar cell layers have the least drug resistance to epirubicin, whereas cell-spheroids have the least resistance to aspirin. Furthermore, spheroids have a slower apoptotic rate and a higher drug resistance, especially for a <50% relative drug concentration. These findings exhibit the chemoresponsive difference between cell-layers and cell-spheroids and further reveal the role of drug diffusivity in the spheroid-body as a non-negligible factor in the cancer chemotherapy. Altogether, further development of this microfluidic device will support the ongoing studies on cancer research, including the chemo-resistance of cancer cell clusters for drug development.

## Supplementary Materials

The following are available online at www.mdpi.com/2072-666X/8/6/167/s1, Figure S1: Apoptosis rates of cells cultured in well plates treated with different concentrations of paclitaxel, epirubicin and aspirin for 4 h, 8 h, 24 h and 30 h of culture durations. Figure S2: Apoptosis rates of cells cultured in well plates treated with different relative concentrations (0–100%) of paclitaxel-aspirin (PA), epirubicin-aspirin (EA) and paclitaxel-epirubicin (PE) mixtures and for 4 h, 8 h, 24 h and 30 h of culture durations. Figure S3: Apoptosis rates of cells cultured in well plates treated with different relative concentrations (0–100%) of a paclitaxel-epirubicin-aspirin (PEA) cocktail and for 4 h, 8 h, 24 h and 30 h of culture durations.
