# Supplementary material for: Investigation of Drug Cocktail Effects on Cancer Cell-Spheroids Using a Microfluidic Drug-Screening Assay
Source: Micromachines (Basel). 2017 May 24;8(6):167. doi: 10.3390/mi8060167 (PMC6189953; doi:10.3390/mi8060167)
Supplement: Supplementary file 1 [file micromachines-08-00167-s001.pdf]

Supplementary Materials: Investigation of Drug Cocktail Effects on Cancer Cell-Spheroids Using a Microfluidic Drug-Screening Assay

Ka I. Au Ieong, Chengpeng Yang, Chin To Wong, Angelie C. Shui, Tom T. Y. Wu, Ting-Hsuan Chen and Raymond H. W. Lam

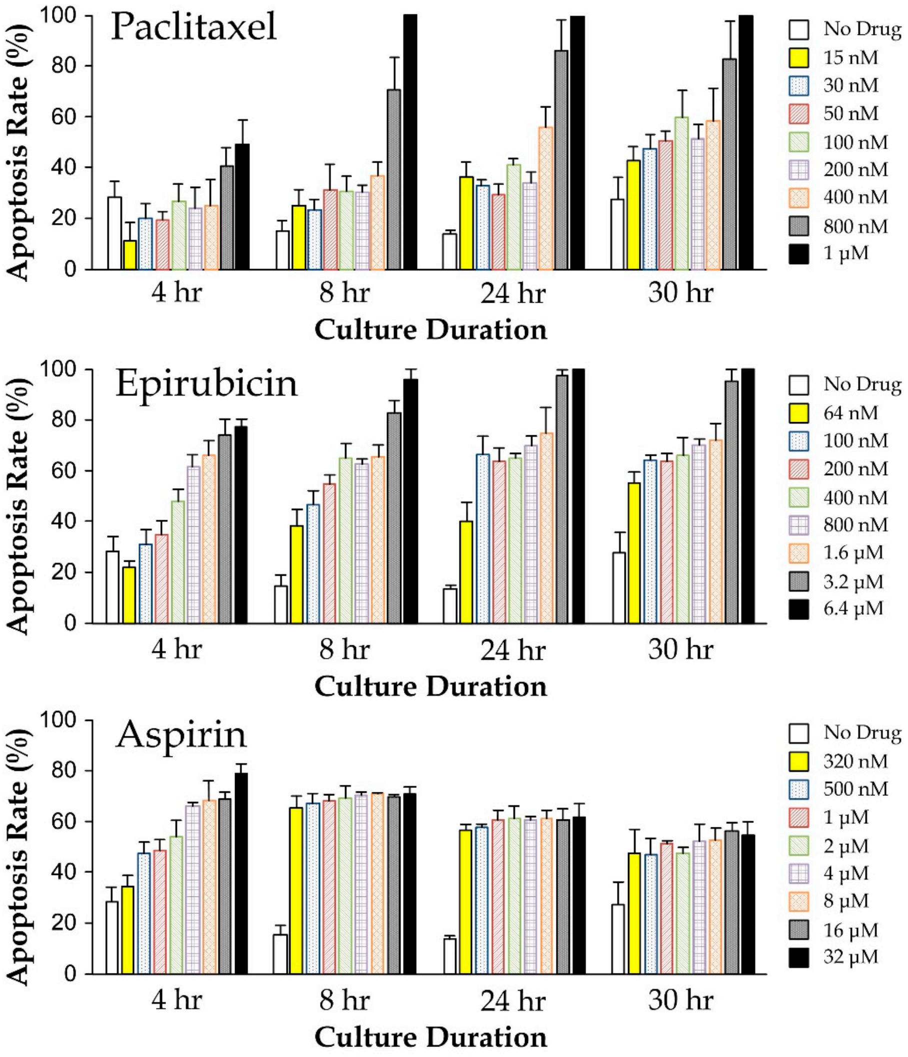

Figure S1. Apoptosis rates of cells cultured in well plates treated with different concentrations of paclitaxel, epirubicin and aspirin for 4 h, 8 h, 24 h and 30 h of culture durations.

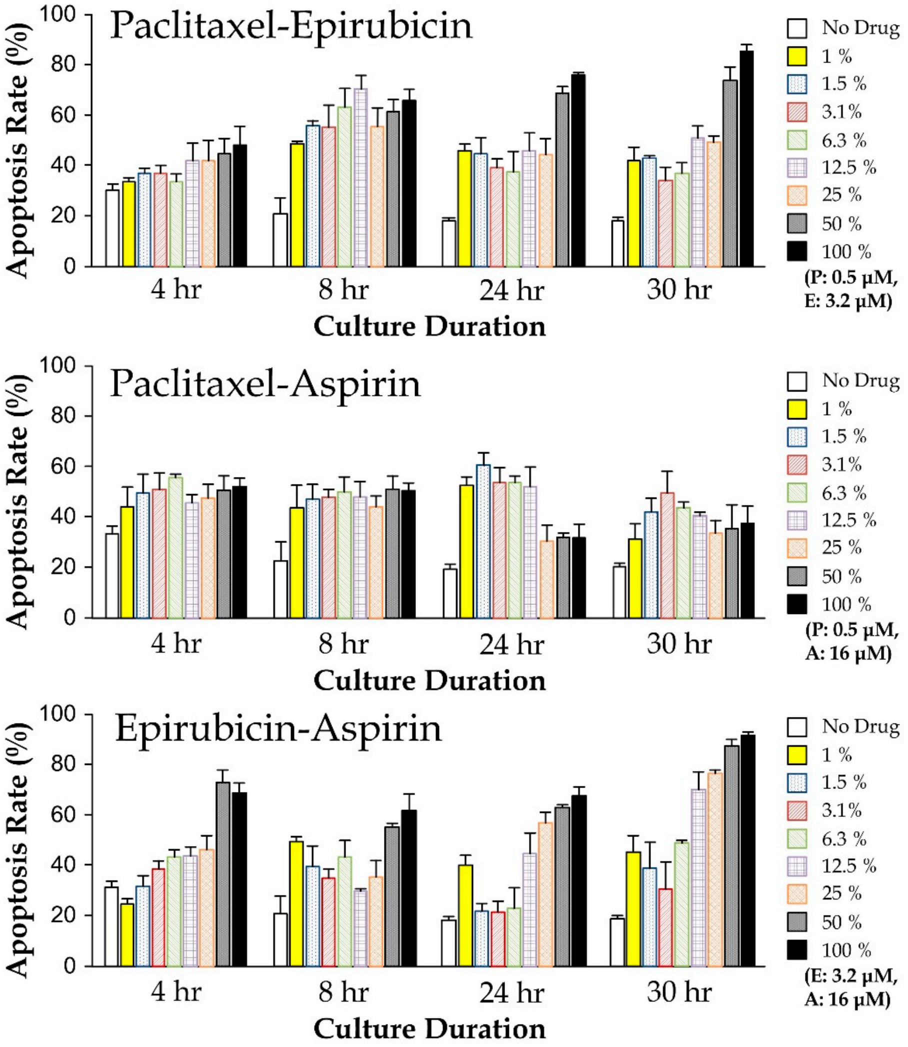

Figure S2. Apoptosis rates of cells cultured in well plates treated with different relative concentrations (0–100%) of paclitaxel-aspirin (PA), epirubicin-aspirin (EA) and paclitaxel-epirubicin (PE) mixtures and for 4 h, 8 h, 24 h and 30 h of culture durations.

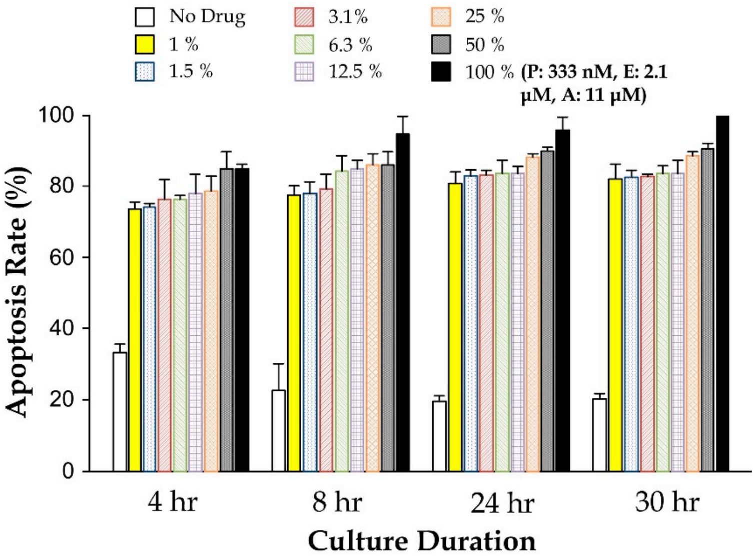

Figure S3. Apoptosis rates of cells cultured in well plates treated with different relative concentrations (0–100%) of a paclitaxel- epirubicin -aspirin (PEA) cocktail and for 4 h, 8 h, 24 h and 30 h of culture durations.
